# High-Throughput Preparation of Silk Fibroin Nanofibers by Modified Bubble-Electrospinning

**DOI:** 10.3390/nano8070471

**Published:** 2018-06-27

**Authors:** Yue Fang, Lan Xu, Mingdi Wang

**Affiliations:** 1 National Engineering Laboratory for Modern Silk, College of Textile and Engineering, Soochow University, 199 Ren-ai Road, Suzhou 215123, China; yfang5279@stu.suda.edu.cn; 2 School of Mechanical and Electric Engineering, Soochow University, 178 Ganjiang Road, Suzhou 215021, China

**Keywords:** silk fibroin, bubble-electrospinning, nanofibers, high-throughput

## Abstract

As a kind of natural macromolecular protein molecule extracted from silk, silk fibroin (SF) has been widely used as biological materials in recent years due to its good physical and chemical properties. In this paper, a modified bubble-electrospinning (MBE) using a cone-shaped gas nozzle combined with a copper solution reservoir was applied to obtain high-throughput fabrication of SF nanofibers. In the MBE process, sodium dodecyl benzene sulfonates (SDBS) were used as the surfactant to improve the spinnability of SF solution. The rheological properties and conductivity of the electrospun SF solutions were investigated. And the effects of gas flow volume, SF solution concentration and additive amounts of SDBS on the morphology, property and production of SF nanofibers were studied. The results showed the decrease of gas flow volume could decrease the nanofiber diameter, enhance the diameter distribution, and increase the production of nanofibers. And the maximum yield could reach 3.10 g/h at the SF concentration of 10 wt % and the SDBS concentration of 0.1 wt %.

## 1. Introduction

Silk fibroin (SF), a kind of natural protein derived from silkworm cocoons, is a widely used as one of the most popular materials for biomedical applications due to its distinct biological properties [1,2], such as good biocompatibility, biodegradability, good cell adhesion and non-toxicity [3,4]. In recent years, many researches have shown that SF biomaterials can be applied widely in tissue engineering scaffolds [5,6], blood vessel tissue engineering [7], bone tissue engineering [8], drug delivery systems, and so on [9,10,11,12]. Through being dissolved and purified, SF obtained from degummed silk can be used to prepare a variety of biomaterials, such as membranes, gels and fibers. Among the forms of biomaterials, SF nanofiber membranes fabricated by electrospinning have great potential for biomedical applications because of their high ratio of surface area and superior mechanical properties [13].

Electrospinning is the most widely used technology for manufacturing nanofibers currently due to its simplicity and cost-efficiency [14,15,16]. The nanofibers produced by electrospinning have excellent characteristics such as small diameter and uniform size distribution. Electrospun SF nanofibers have attracted much attention in the field of tissue engineering [17,18]. Nalvuran et al. [17] used electrospinning to obtain nanofibrous SF/reduced graphene oxide scaffolds for tissue engineering and cell culture applications. Brito-Pereira et al. [6] prepared SF-magnetic hybrid composite electrospun fibers for tissue engineering applications. And Yu et al. [18] developed a new electrospinning approach for fabricating biomimetic thermoplastic polyurethane/SF small-diameter vascular grafts. However, the conventional single-needle spinning has a very fatal weakness: low production, usually at the level of 0.01–0.1 g/h, which leads to inhibit the industrial application of nanofibers [19].

Bubble-electrospinning (BE) is one of the most effective free surface electrospinning for high-throughput preparation of nanofibers [20,21,22,23,24,25,26]. It is well known that the applied voltage is a very crucial parameter affecting the quality and production of nanofibers [24,27]. However, the BE setup consists of a metal electrode that is fixed in the polymer solution reservoir and connected to the power generator. The metal electrode leads to lower applied voltage, usually about 30 kV, which results in low-throughput fabrication of nanofibers [27]. Therefore, a modified bubble electrospinning (MBE) was presented to obtain high-throughput fabrication of high quality nanofibers in our previous works [22,27]. The MBE apparatus was developed and used firstly to enhance the production of quality PVA nanofibers, and its mechanism was studied experimentally and theoretically [27]. Secondly, the MBE apparatus combined with a high speed rotating copper wire drum as a collector, was presented successfully to obtain high throughput preparation of aligned PAN nanofibers [22]. In these studies, high throughput production of nanofibers could be easily obtained by MBE under a high applied voltage up to 70 kV.

**Figure 1:**
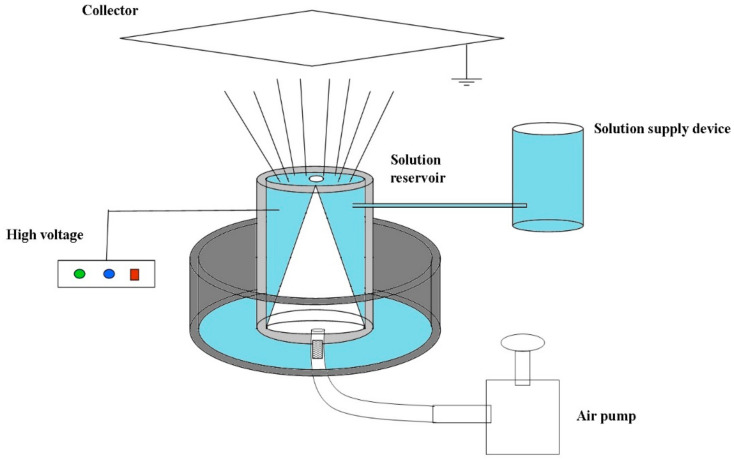
The schematic presentation of MBE apparatus.

Figure 1 shows the schematic presentation of the MBE apparatus. Compared to BE, the MBE using a cone-shaped polymer nozzle combined with a copper solution reservoir could produce nanofibers under a much higher applied voltage which would improve the quality and production of nanofibers.

In this study, the MBE was applied to obtain high-throughput preparation of silk fibroin nanofibers. However, the large surface tension of the regenerated SF solution would lead to bad spinnability of the solution in the MBE process. Sukigara et al. [28] and Geng et al. [29] proposed that when the SF solution concentration is too high the viscosity and surface tension are too large, and the SF solution will not be favorable for spinning. Cai et al. also mentioned that the surface tension of 27.5 wt % SF aqueous solution could reach 33.8 mN/m [30]. Surface-active agents are generally used to decrease the surface tensions of polymer solutions [31]. Therefore, an anionic surfactant, sodium dodecylbenzene sulfonate (SDBS) had been used to reduce the surface tension of the spinning solution in order to improve the spinnability of SF solution in the MBE process [32,33]. The rheological properties and conductivity of the electrospun solutions were investigated by rheometer and conductivity meter. And the effects of gas flow volume, elecrospun SF solution concentration and additive amounts of SDBS on the property and production of SF nanofibers were studied by scanning electron microscopy (SEM), universal electromechanical test machine, precise electronic balance, and other methods.

## 2. Materials and Methods

### 2.1. Materials

Bombyx mori silk (Cangzhou Xiehe Silk Co., Ltd., Cangzhou, China) was boiled three times in 0.5% NaCO_3_ solution for 0.5 h each time [34]. After washing with distilled water several times, the degummed silk fibers were dissolved in 80 wt % lithium bromide (Alfa Aesar, Shanghai, China) at 60 °C for 4 h, and then dialyzed against distilled water in a cellulose tube (Viskase, 7000 Da, Chicago, IL, USA) at room temperature for 3 days. After the aqueous solution was centrifuged twice at 4000 r/s for 20 min. (H1850, Hunan Xiangyi Experimental Instrument Development Co., Ltd., Changsha, China), a SF aqueous solution was obtained at a concentration of 4–6 wt %. The SF solution was poured into a petri dish with 10 cm diameter, and placed in an oven for drying at 40 °C for 36 h. Finally, the SF membrane was obtained. The membrane was packed in a clean sealed bag, and was broken by hand. Then the membranes shredded were poured into a mortar and pulverized into powders.

The SF powders were dissolved in formic acid (98%, Yonghua Chemical Technologe Co., Ltd. Jiangsu, China) together with sodium dodecyl benzene sulfonate (SDBS) (Sinopharm Chemical Reagent Co., Ltd., Shanghai, China). The solution was stirred on the magnetic stirrer (HJ-6A, Gongyi Yuhua Instrument Co., Ltd., Gongyi, China) and dissolved for 8 h to prepare the solution shown in Table 1. All the reagents were of analytical reagent grade and used without further purification, and all concentration measurements were done in weight per weight (w/w). SF and SDBS concentration were related to the spinning solution.

### 2.2. MBE Process

According to References [22,24,27,35], the electrospinning parameters were set as follows: SF concentration varied from 6 to 10 wt %, SDBS concentration varied from 0.1 to 0.7 wt %, the gas flow volume varied from 50 to 150 m^3^/h, the applied voltage was 50 kv, and the working distance from the nozzle to the grounded collector was 18 cm. The MBE experiments were carried out at room temperature (20 °C) and at a relative humidity of 60%.

**Figure 2:**
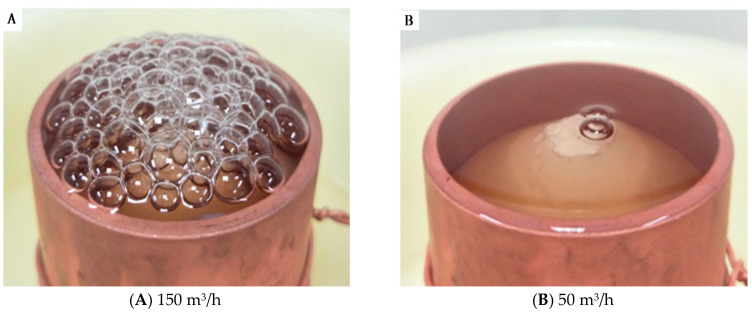
Photograph of the MBE with different gas flow volume of (**A**) 150 m^3^/h and (**B**) 50 m^3^/h, respectively.

The SF solution was poured into the reservoir, and the distance from the top of the nozzle to the SF solution surface was 5 mm. At the same time, turning on slowly the gas valve and high-voltage power generator, a stream of gas was injected into the spinning solution through a conical nozzle. When the gas flow volume was 50 m^3^/h (B), the whole solution surface became convex. And when the gas flow volume was 150 m^3^/h (A), bubbles were generated at the SF solution surface (see Figure 2). After an electronic field was applied and the surface tension of the convex solution surface or the bubbles was overcome by the electric field force, multiple jets initiated and were stretched and refined to form SF nanofibers which were received on the collector eventually.

### 2.3. Measurement and Characterization

Diameter and arrangement of electrospun SF nanofibers were characterized using a scanning electron microscopy (Hitachi S-4800, Hitachi, Tokyo, Japan). All samples were dried at room temperature and then sputter-coated with gold by an E-1045 (Hitachi, Tokyo, Japan) for 90 s. The matrix morphology and fibrous diameter characterization were carried out using Image J software (National Institute of Mental Health, Bethesda, MD, USA). To determine the diameter distribution of nanofibers, 50 SEM images and 100 nanofibers at random in each SEM image were chosen for diameter distribution analysis by ImageJ software.

Rheological studies of SF solutions were conducted by a Rheometer (AR2000, TA Instruments, New Castle, DE, USA) with a 40 mm cone plate (Ti, 40/1°). The normal force applied on the sample during lowering of the top plate was limited to 0.1 N. The shear rate was linearly increased from 0.1 to 1000 1/s at 25 °C. All rheological measurements were repeated two times.

The conductivities of SF solutions were determined by conductivity meter (DDS-307A, Shanghai INESA Analytical Instrument Co., Ltd. ShangHai, China) at room temperature. The test samples were divided into two groups, namely the pre-spinning solution and the spinning solution collected after spinning for half an hour. The measurement was repeated five times.

FTIR spectra of SF nanofiber membranes (NFMs) were obtained on a Fourier transform infrared (FTIR) spectroscopy (Nicolet5700, Thermo Nicolet Company, Waltham, MA, USA) by the performance of 32 scans with the wave number ranging from 400 to 4000 cm^−1^ with a resolution of 4 cm^−1^. First 1–2 mg of NFMs shredded was mixed with about 200 mg of KBr powder. Then the mixture was pressed into a tablet which was then used in the analysis.

The mechanical properties of SF NFMs reported in Table 1 were all carried out by a universal electromechanical test machine Instron-3365 (Instron, Norwood, MA, USA). All samples were 40 mm × 10 mm rectangle membranes. The test conditions were a clamping length of 20 mm, a pre-tension of 0.2 cN and a tensile rate of 100 mm/min, respectively. Before performing the mechanical tests, these NFMs were placed in a constant temperature and constant pressure chamber and equilibrated for 24 h to achieve their moisture balance and stabilize the beta-sheet structure of SF. The measurement was repeated five times.

The contact angle (CA) measurements of SF NFMs were performed with a Krüss K100 apparatus (Krüss Company, Hamburg, Germany). The volume of droplet used for static CA was 6 mL. Moreover, the average water CA of each NFM were obtained by measuring five different positions of the same NFM. The five samples with a flat surface cut from the same NFM were 20 mm × 20 mm square membranes, and their four corners were pressed by a glass slide to eliminate the influence of their surface morphology on the test results.

## 3. Results and Discussion

### 3.1. Rheological Property

**Figure 3:**
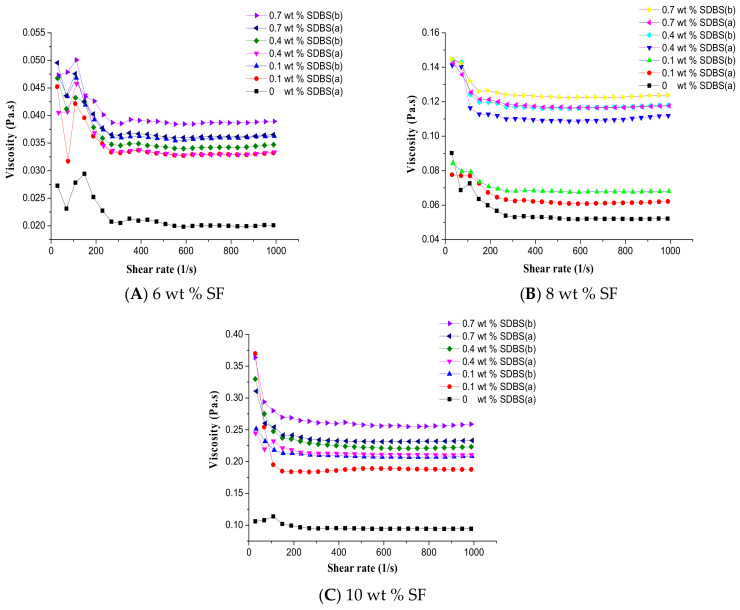
Rheological behavior between the shear viscosity and shear rate of the spinning solutions before spinning (a) and after spinning for 30 min (b) with SF concentrations of (**A**) 6 wt %; (**B**) 8 wt % and (**C**) 10 wt %, respectively.

The effects of SF concentration, SDBS content and spinning time on the rheological properties of the solutions were investigated. Figure 3 displayed the rheological behavior between the shear viscosity and shear rate of SF solutions with different concentrations of SDBS before (a) and after (b) spinning. It could be seen that the viscosity of the spinning solution increased slightly after spinning for 30 min. It was because that the MBE apparatus was placed in an open environment, and the solvent of the spinning solution was volatile formic acid. With the increase of spinning time the viscosity of the spinning solution increased slightly due to the volatilization of solvent. The viscosity increased could influence slightly the fiber diameters in a same membrane. And the uniformity of the SF nanofibers could be decreased slightly.

In addition, Figure 3 also illustrated with the increase of SF concentration and SDBS content the viscosity of the spinning solution showed an increasing trend. To the best of our knowledge, the viscosity of solutions increased with the increase of the solution concentration [36]. And it’s reported [37] that the fluid shear thinning behavior was enhanced with the increase of solution concentration, and the fluid shear thinning behavior was mainly caused by the orientation of macromolecular chains. With the increase of shear rate, the number of the oriented macromolecular chains increased, which could decrease the viscosity and enhance the shear thinning behavior. At the same time, it could be seen from Figure 3 that the addition of SDBS resulted in the significant increase of the spinning solution viscosity, and the viscosity showed an increasing trend with the increase of SDBS concentration. It could be because that the silk fibroin molecules were positively charged in the spinning solution, while the anionic surfactant SDBS was negatively charged. The negatively charged SDBS was bound to the positively charged SF macromolecular chain as a pendant group, which led to the increase of the steric hindrance between the molecular chains, so the solution viscosity increased.

### 3.2. Conductivity of Solutions

**Figure 4:**
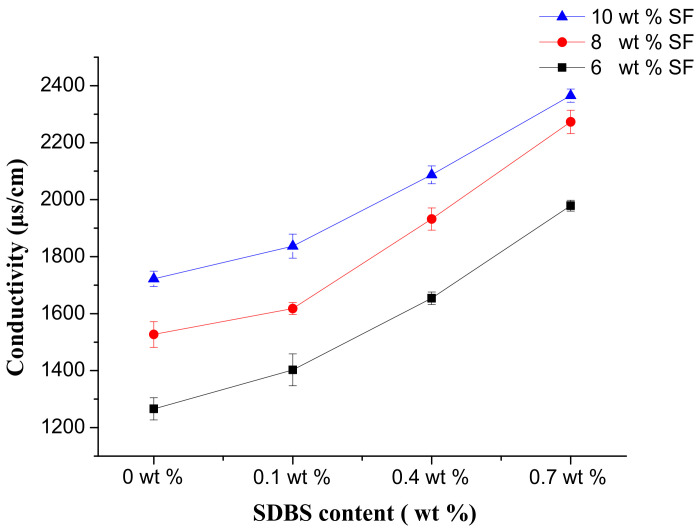
Conductivity of the spinning solutions.

Figure 4 showed the conductivity of the spinning solution with the different concentration of SF. It could be found that the conductivity increased with the increase of SF concentration. And as the concentration of SDBS increased, the conductivity of the solution also increased linearly. This was because the conductivity reflected the concentration of conductive particles—sodium ions in the spinning solution. The higher the concentration of conductive particles, the higher the conductivity would be. As the concentration of SF and SDBS increased, the concentration of conductive particles in the solution increased, and the conductivity of solution showed an increasing trend.

### 3.3. Morphology Characterization of SF Nanofibers

**Figure 5:**
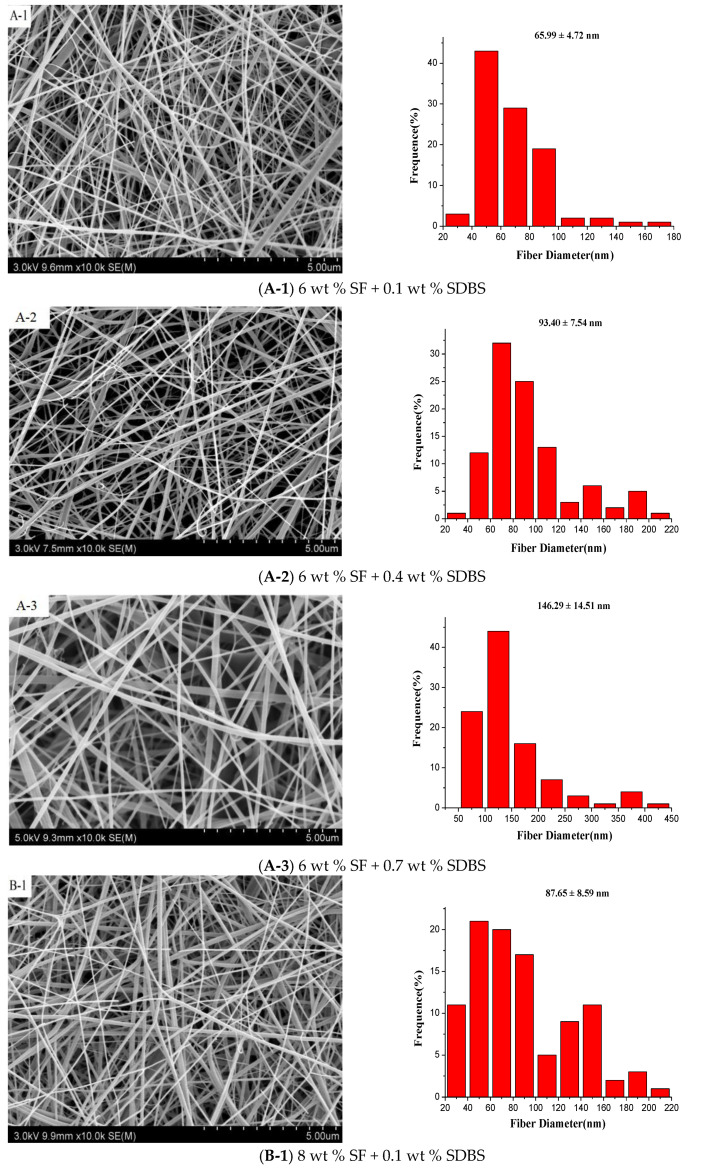

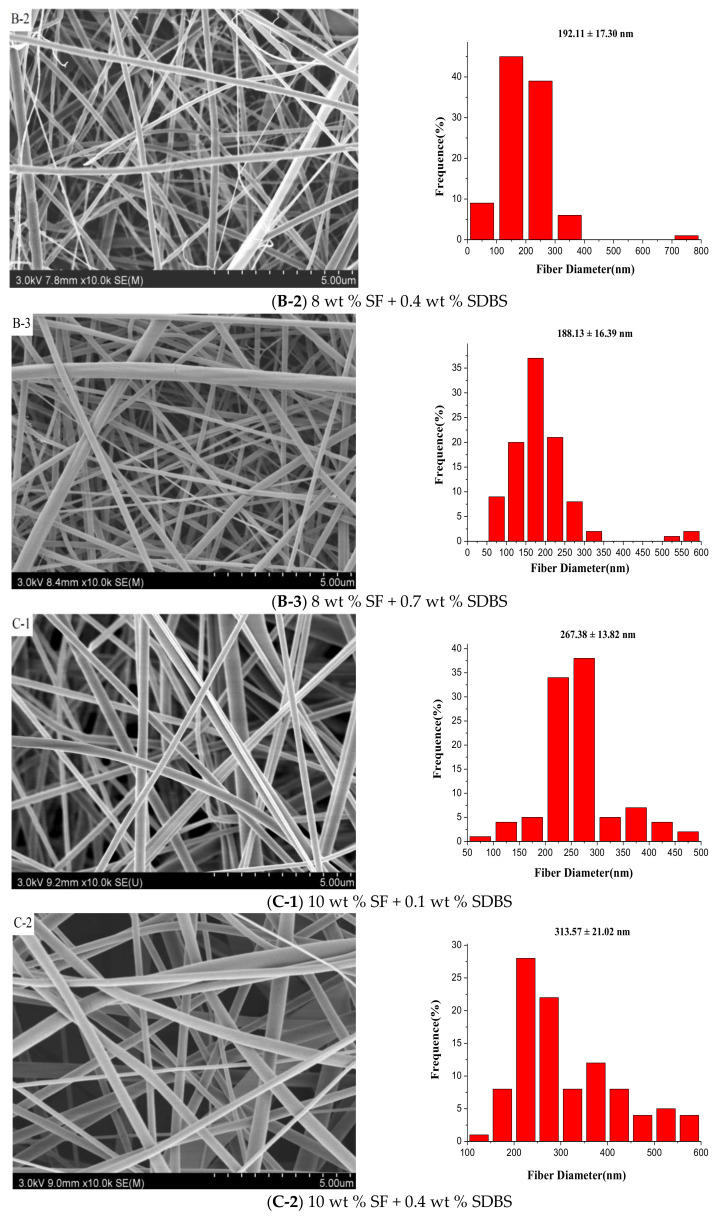

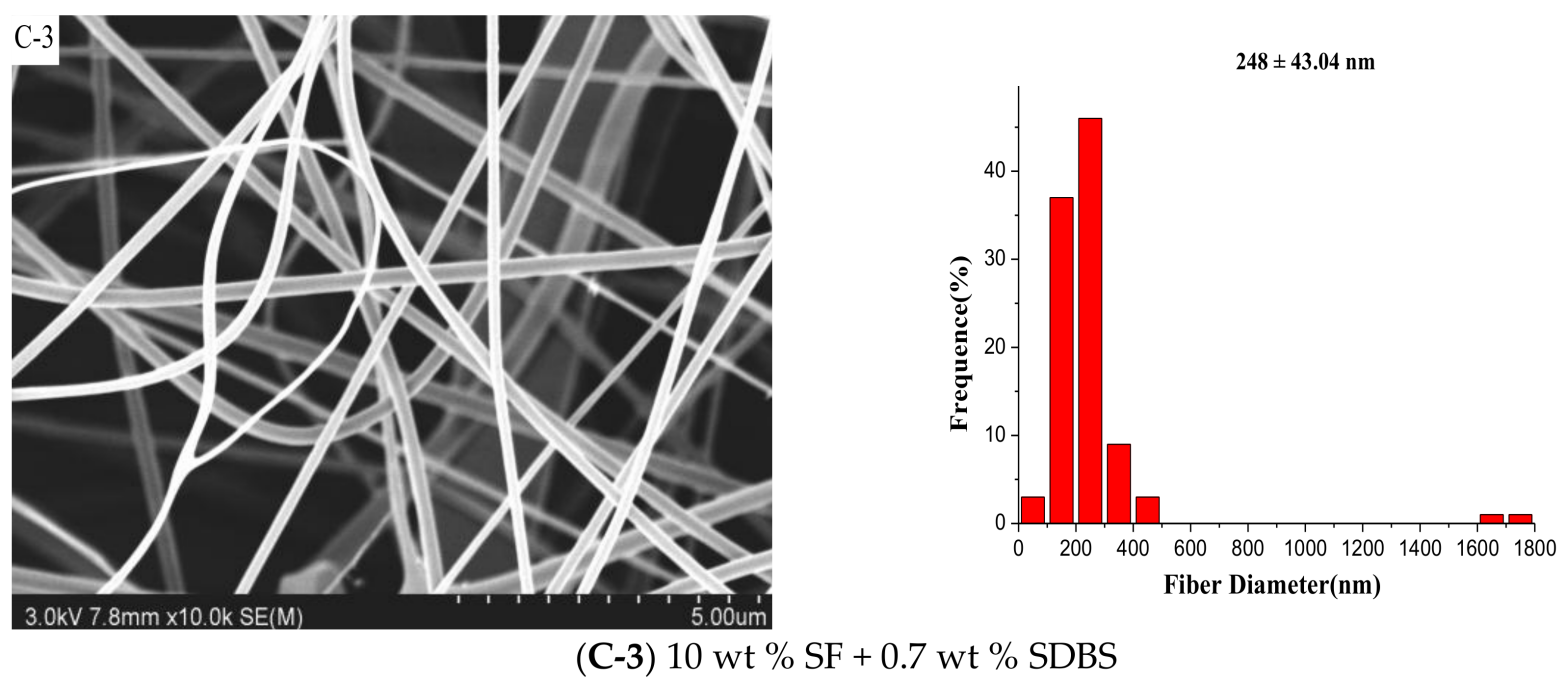
SEM pictures and the according diameter distribution of the SF nanofibers with SF concentrations of (**A**) 6 wt %; (**B**) 8 wt % and (**C**) 10 wt %, respectively. (Gas flow volume: 150 m^3^/h)

**Figure 6:**
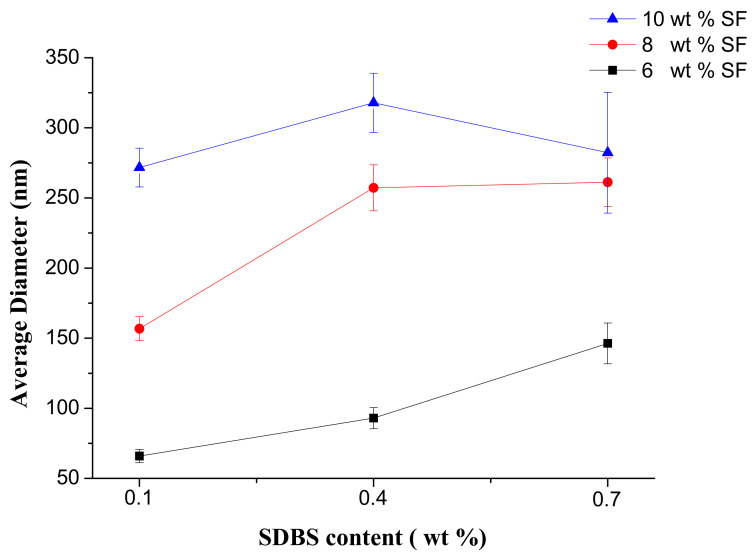
Average diameter of SF nanofibers when the gas flow volume was 150 m^3^/h.

Effects of gas flow volume, SF concentration, SDBS content on the morphology of SF nanofibers were investigated respectively by SEM. Figure 5 showed the SEM pictures and the according diameter distribution of SF nanofibers with the different SF concentrations and SDBS contents when the gas flow volume was 150 m^3^/h. And Figure 6 and Table 2 indicated the effects of SF concentration and SDBS content on the average value, standard deviation value and confidence interval of SF nanofiber diameter, respectively. The standard deviation values were high due to measuring nanofiber diameters by observed sample data. Therefore, a confidence interval gave an estimated range of values which was likely to include unknown diameters of fibers. The estimated range was calculated from a given set of sample data [38], and the confidence intervals obtained were presented in Table 2.

It was evident that as the SF concentration increased the average value, standard deviation value and confidence interval of SF nanofiber diameter increased. It was because with the increase of the viscosity, the polymer chains would inhibit electric field stretching, and the nanofiber diameters increased. And when the SF solution concentrations were 6 wt % and 8 wt %, the average value, standard deviation value and confidence interval of SF nanofiber diameter increased with SDBS concentration increasing due to the solution viscosity increased. But when the SF solution concentration was 10 wt %, the average diameter of SF nanofibers increased firstly and then decreased, and standard deviation value and confidence interval of these increased, which could be related to the combined effects of the viscosity and conductivity of the spinning solution [39,40]. Moreover, it was difficult to collect nanofibers when the SDBS concentration is 0.7 wt %, see Figure 5. It was because when the conductivity was too large, the corresponding repulsion of charged jet was too large, which would make it very difficult to collect nanofibers. As a result, the average value and confidence interval of SF nanofiber diameter was smallest at the SF concentration of 6 wt % and the SDBS concentration of 0.1 wt %.

**Figure 7:**
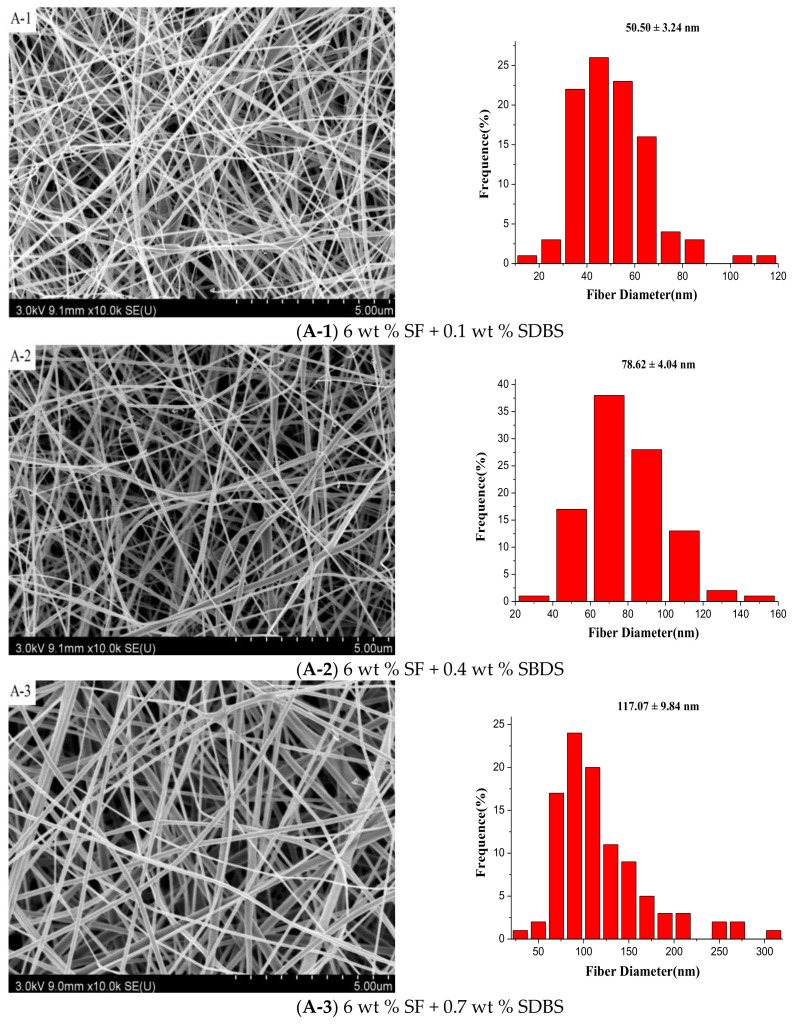

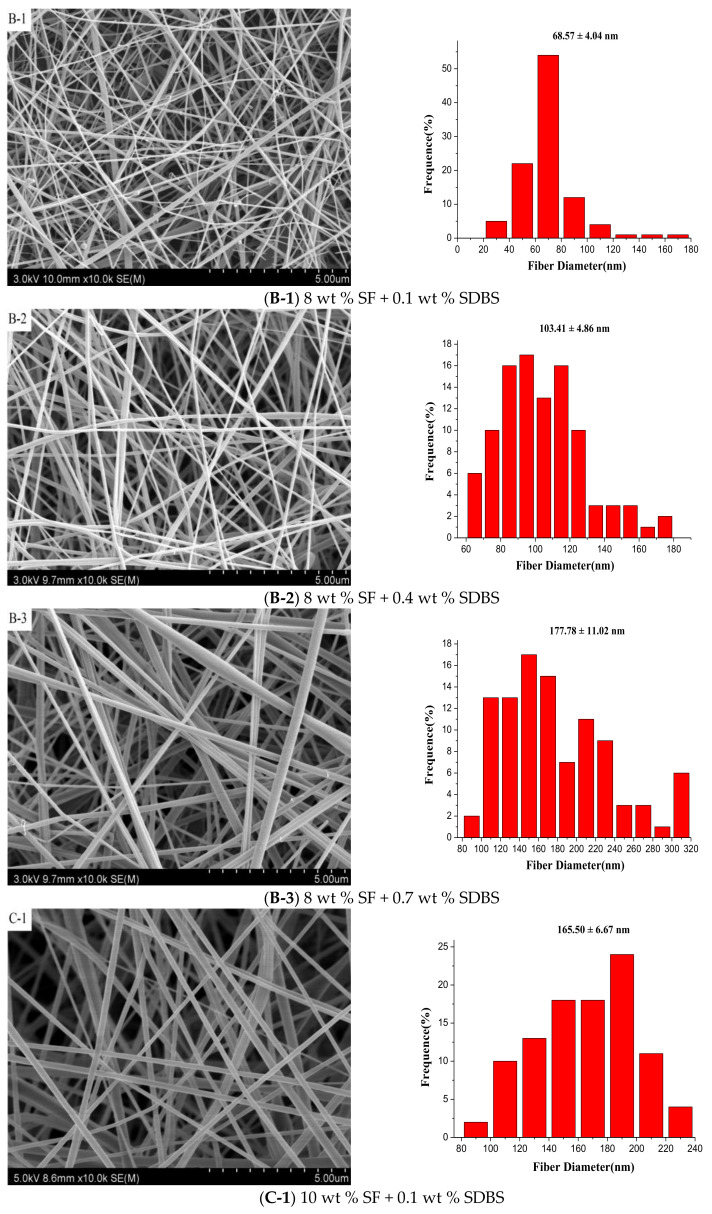

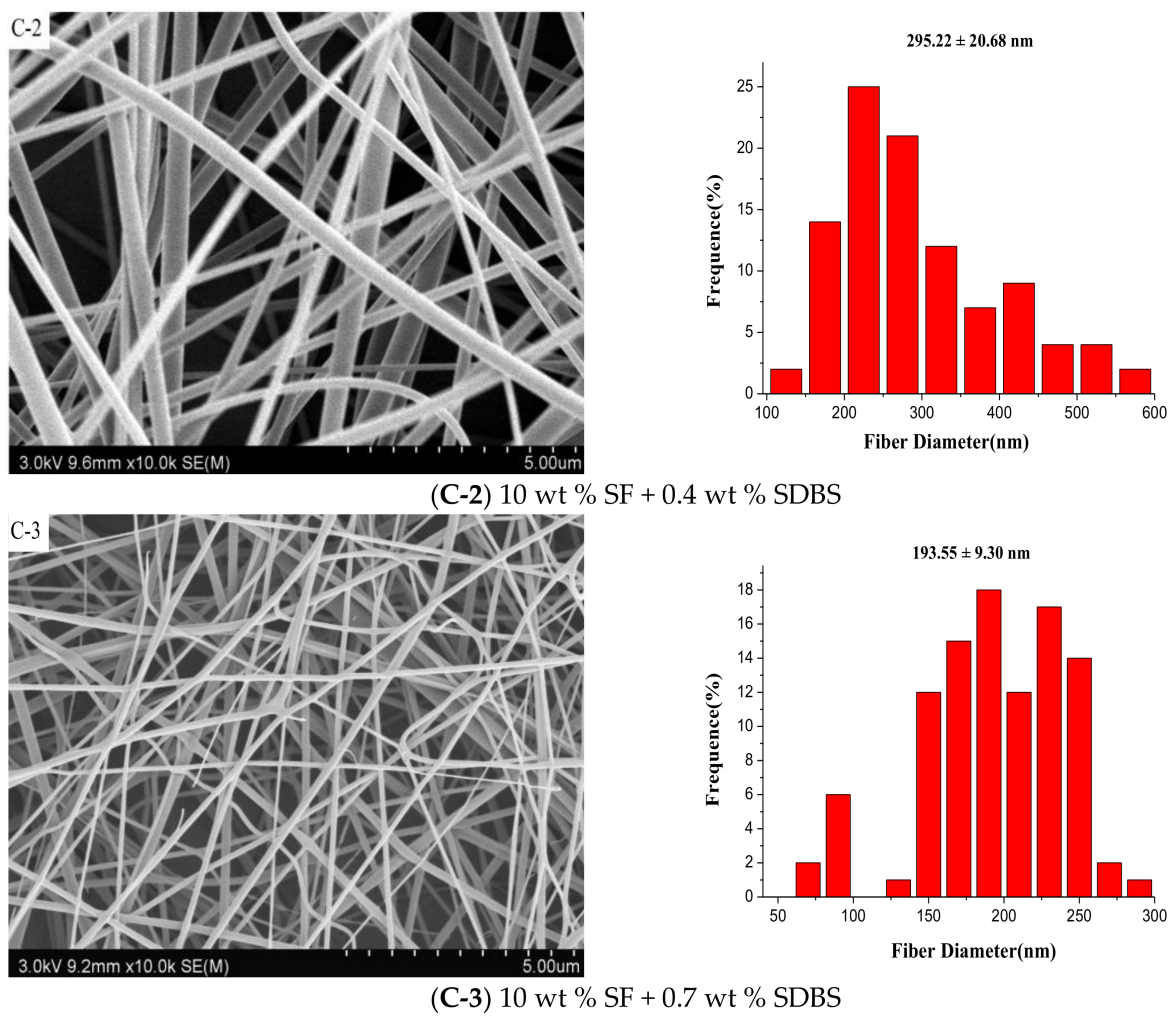
SEM pictures and the according diameter distribution of the SF nanofibers with SF concentrations of (**A**) 6 wt %; (**B**) 8 wt % and (**C**) 10 wt %, respectively. (Gas flow volume: 50 m^3^/h)

**Figure 8:**
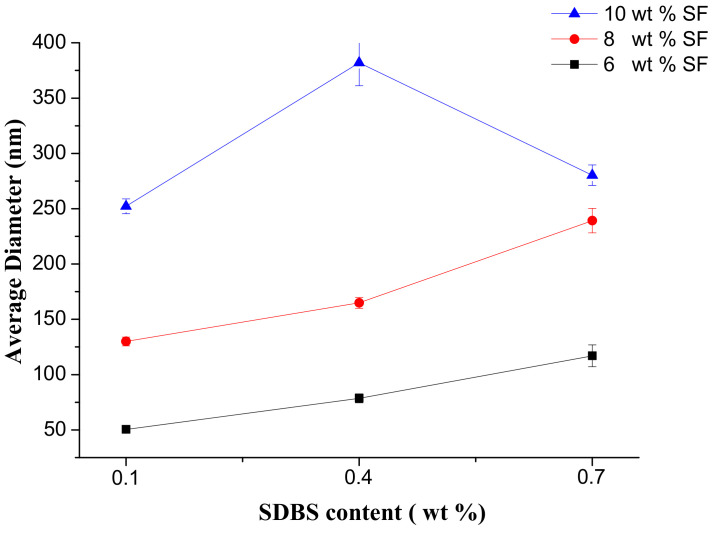
Average diameter of SF nanofibers when the gas flow volume was 50 m^3^/h.

Figure 7 illustrated the SEM pictures and the according diameter distribution of SF nanofibers with the different SF concentrations and SDBS contents when the gas flow volume was 50 m^3^/h. And Figure 8 and Table 3 showed the effects of SF concentration and SDBS contents on the average value, standard deviation value and confidence interval of SF nanofiber diameter, respectively. It was obvious to see that with the change of SF concentration and SDBS contents the average value, standard deviation value and confidence interval of SF nanofiber diameter displayed almost the same change trend as when the gas flow volume was 150 m^3^/h. However, it could be found that when the gas flow volume decreased, the average diameter of SF nanofibers decreased, and the diameter distribution was more uniform. It was because when the gas flow volume was 150 m^3^/h, the generated bubbles would increase the nanofiber diameter and reduce the uniformity of diameter distribution due to bubble formation, deformation and break wasted energy which could be used to further stretch the jet into smaller fibers [27]. Therefore, the gas flow volume of 50 m^3^/h was selected as the optimal parameter for spinning, and the properties and yields of the NFMs produced in this situation were characterized. And when the SF concentration was 6 wt % and the SDBS concentration was 0.1 wt %, the average value and confidence interval of SF nanofiber diameter was smallest. It was reported that SF/formic acid solution could be used to produce nanofibers with diameters of 50 to 300 nm by electrospinning [41]. Compared to the electrospinning, the diameters of SF nanofibers prepared by MBE had the same range.

### 3.4. Fourier-Transform Infrared (FTIR) Spectroscopy

**Figure 9:**
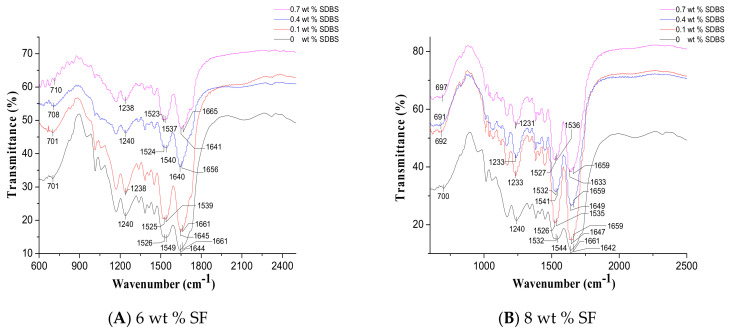

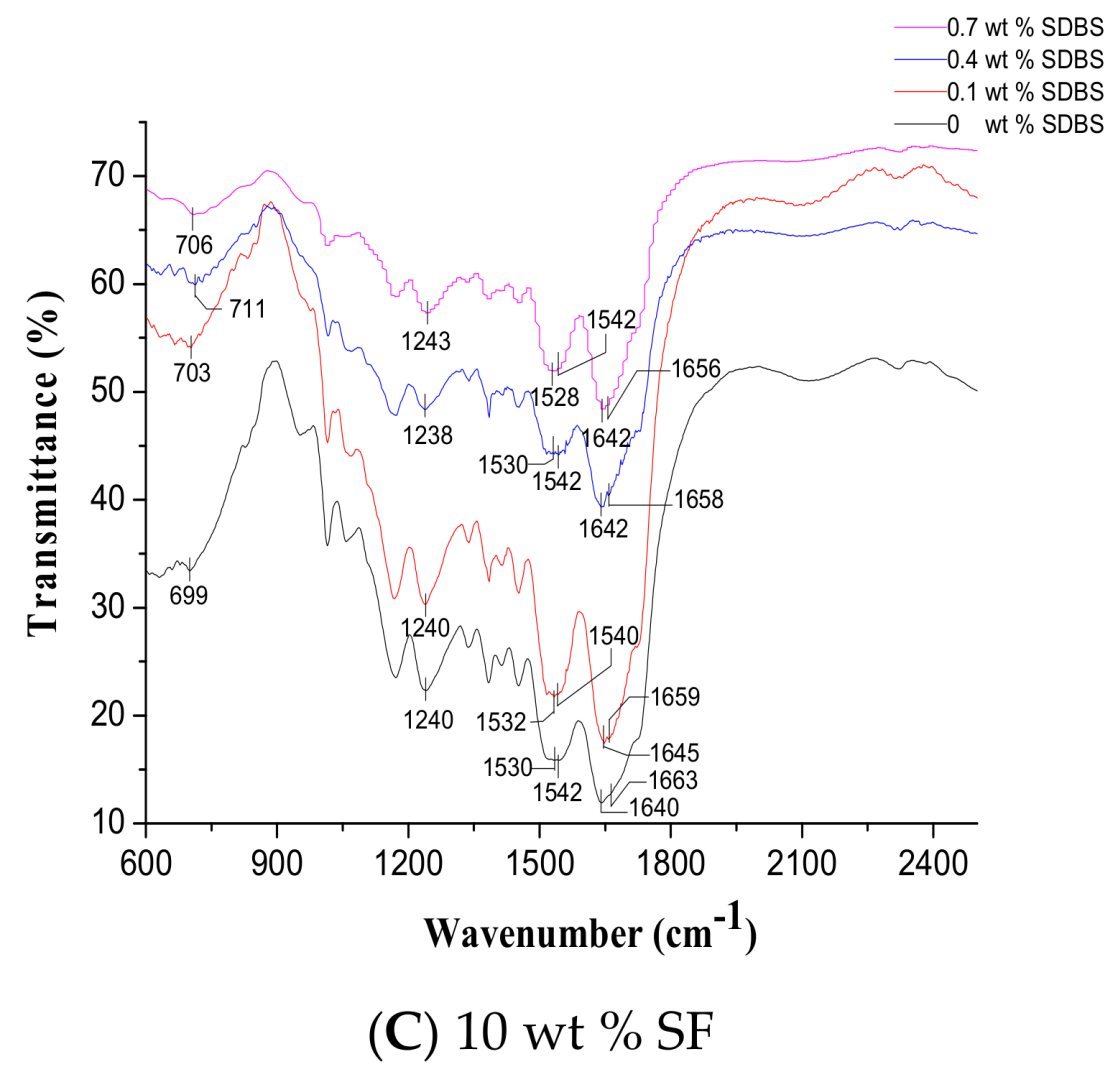
FTIR spectra of NFMs with SF concentrations of (**A**) 6 wt %; (**B**) 8 wt % and (**C**) 10 wt %, respectively. (Gas flow volume: 50 m^3^/h).

It was reported that the characteristic absorption bands of SF appeared at 1630 cm^−1^ (amide I), 1530 cm^−1^ (amide II), 1265 cm^−1^ (amide III) and 700 cm^−1^ (amide Ⅴ) were assigned to the *β*-sheet form (silk II), while the bands at 1660 cm^−1^ (amide I), 1540 cm^−1^ (amide II), 1235 cm^−1^ (amide III) and 650 cm^−1^ (amide V) were attributed to the random-coil and α-helix form (silk I) [42,43,44]. Figure 9 showed the FTIR spectra of NFMs with the different SF concentrations and SDBS contents. It was obvious that the characteristic absorption peaks at around 1642 cm^−1^ (amide I), 1527 cm^−1^ (amide II) and 701 cm^−1^ (amide V) corresponded to silk II were observed, and the peaks at around 1660 cm^−1^ (amide I), 1540 cm^−1^ (amide II) and 1237 cm^−1^ (amide III) assigned to silk I were also observed [42]. Figure 9 also indicated little change in the spectra of NFMs with the increase of SDBS contents. Moreover, there were no new characteristic peaks after the addition of SDBS, probably because of the very low ratio of SDBS with respect to SF. In future, these SF NFMs will be treated with methanol solution to stabilize the *β*-sheet conformation of SF (silk II).

### 3.5. Mechanical Properties of SF Nanofiber Membrane

**Figure 10:**
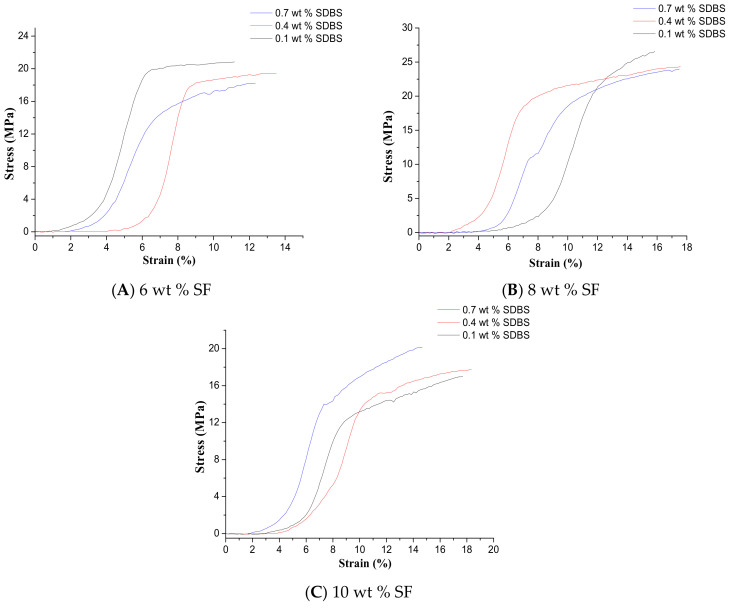
Stress-strain curves of NFMs of the SF nanofibers with SF concentrations of (**A**) 6 wt %; (**B**) 8 wt % and (**C**) 10 wt %, respectively. (Gas flow volume: 50 m^3^/h).

The mechanical properties, such as breaking strength and elongation at break, of NFMs with the different SF concentrations and SDBS contents, were presented in Table 4. And a stress-strain curves of these NFMs was shown in Figure 10. It could be seen that with the increase of SF concentrations the tensile strength of NFMs firstly increased and then decreased, and the elongation at break of NFMs increased. The SEM pictures of these NFMs in Figure 7 showed that as the SF concentration increased the average diameter of SF nanofibers increased, indicating that the weakened mechanical properties were due to too small or too large nanofiber diameter. Therefore, NFMs with the SF concentration of 8% exhibited stronger mechanical performance due to the moderate nanofiber diameter. However, the standard deviation of mechanical performances was largest in this situation. It could be because the thickness of NFM was not equal due to uneven distribution of nanofibers. When SF concentration was 6 wt % and 8 wt % the tensile strength of NFMs decreased with increasing SDBS, and the elongation at break of NFMs increased. And when SF concentration was 10 wt % the tensile strength of NFMs increased with increasing SDBS, and the elongation at break of NFMs decreased. Considering the average value of tensile strength and elongation at break combined with standard deviation, it could be found that when SF concentration was 10% and SDBS content was 0.7% the mechanical properties of NFMs were best relatively due to the even NFM thickness, which could be related to the combined effects of the viscosity and conductivity of the spinning solution in the MBE process. In future electrospun composite NFMs with excellent mechanical properties compared to the pure SF NFMs would be prepared because nanocomposites exhibited enhanced mechanical properties [45,46].

### 3.6. Contact Angle Measurements

**Figure 11:**
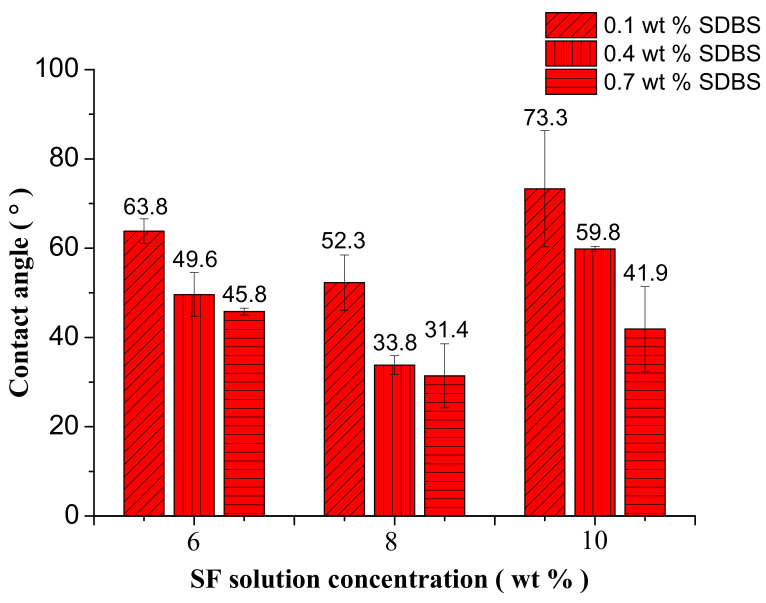
Contact angles of NFMs with the different SF concentrations and SDBS contents when the gas flow volume was 50 m^3^/h.

**Figure 12:**
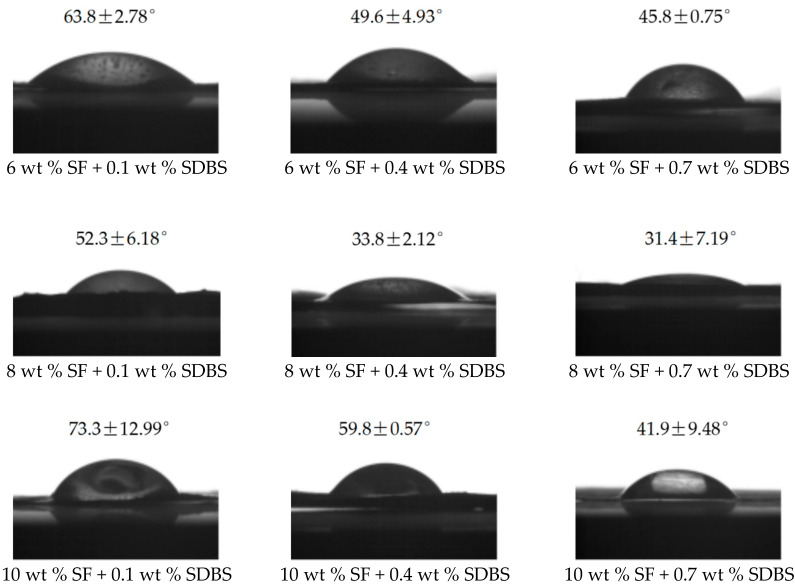
The representative images of the CA measurements.

The contact angle (CA) value of SF NFM prepared from the spinning solution with 10% SF concentration by electrospinning was about 64.2 ± 4.2° reportedly [47]. Figure 11 showed the CA values of electrospun NFMs with the different SF concentrations and SDBS contents, and Figure 12 illustrated the representative images of the CA measurements. It revealed that these NFMs were all hydrophilic materials, and with the increase of SDBS contents, the CA decreased and the hydrophilicity became stronger due to the hydrophilic structure of SDBS. In addition, it could be seen that when SF concentration was 8% the hydrophilicity of NFMs was best due to the uneven NFM thickness as illustrated in Table 4, which made the surface of NFMs rough. It was reported that the rough surface could make hydrophilic materials more hydrophilic [48]. Uneven NFM thickness meant that the surface of NFM obtained from the spinning solution with SF concentration of 8% was rougher than other NFMs. Compared to the electrospinning, the CA values of NFM prepared from the spinning solution with 10% SF concentration by MBE had a larger scope.

### 3.7. Yield of SF Nanofibers

**Figure 13:**
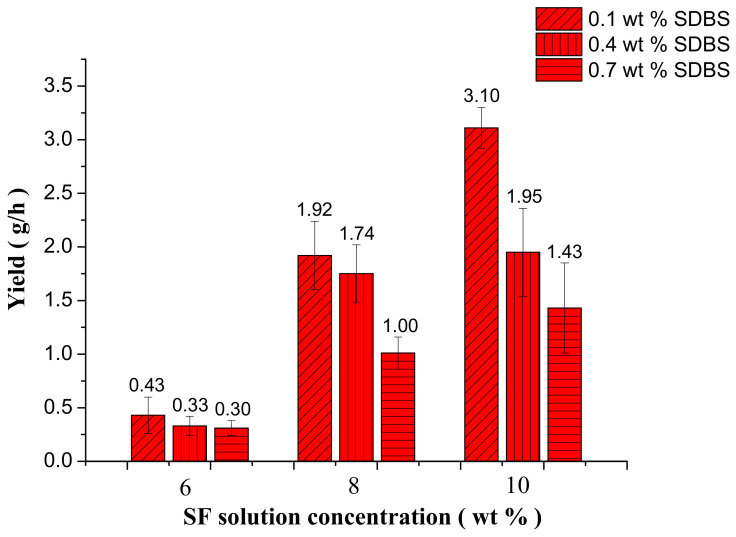
Yield of the SF nanofibers with the different SF concentrations and SDBS contents when the gas flow volume was 50 m^3^/h.

After spinning for 30 min, the mass of SF NFMs produced by MBE was measured by precise electronic balance. Then the yield of SF nanofibers was calculated. Figure 13 illustrated yield of SF nanofibers with the different SF concentrations and SDBS contents. It could be seen that with the increase of SF concentration the yield of SF nanofibers increased due to the volatilization of formic acid in the MBE process. However, with the increase of SDBS content, the yield of SF nanofibers has been decreasing. This might be because that with the increase of SDBS, the surface tension of the solution decreased and more bubbles were generated. The generated bubbles wasted energy which could make the charged jets move faster during the MFSE process, and as a result the yield of SF nanofibers decreased. When SF concentration was 10 wt % and SDBS content was 0.1 wt %, the yield reached maximum value and was 3.10 ± 0.19 g/h. According to literature [19] and our work, the yield of SF nanofibers produced by electrospinning was only about 0.1 g/h. It meant that the MBE could enhance the yield of SF nanofibers 30 times more than the electrospinning.

## 4. Conclusions

In this paper, high-throughput fabrication of SF nanofibers has been obtained by a modified bubble-electrospinning (MBE) using a cone-shaped polymer nozzle combined with a copper solution reservoir. SDBS was added in the SF solutions to improve the solution spinnability, and the rheological properties and conductivity of the spinning solutions were investigated. The results showed with the increase of SF concentration and SDBS content, the viscosity and conductivity of spinning solutions increased.

Then the effects of gas flow volume, SF concentration and additive amounts of SDBS on the morphology of SF nanofibers were studied respectively. The results indicated when the gas flow volume was 150 m^3^/h, the generated bubbles would increase the nanofiber diameter and reduce the uniformity of diameter distribution. And the average value and standard deviation value of SF nanofiber diameters was smallest at the SF concentration of 6 wt % and the SDBS concentration of 0.1 wt %.

Finally, the gas flow volume of 50 m^3^/h was selected as the optimal for, and the mechanical properties, wetting properties and yields of the NFMs produced in this situation were characterized by universal testing machine, Krüss K100 apparatus and precise electronic balance. The results showed the quality and production of nanofibers were improved with the increase of SF solution concentration. Considering the combined effects of spinning parameters on the property and production of SF nanofibers, the optimal MBE conditions were gas flow volume of 50 m^3^/h, SF concentration of 10 wt % and SDBS concentration of 0.1 wt %. And the MBE could enhance the yield of SF nanofibers 30 times more than the electrospinning.

## Figures and Tables

**Table 1:** Composition and concentration of the spinning solution.

SF (wt %)	6			8			10		
**SDBS (wt %)**	0.1	0.4	0.7	0.1	0.4	0.7	0.1	0.4	0.7
**No.**	1	2	3	4	5	6	7	8	9

**Table 2:** Effects of SF concentration and SDBS content on the average diameter of SF nanofibers when the gas flow volume was 150 m^3^/h.

SF (wt %)		6			8			10		
SDBS (wt %)		0.1	0.4	0.7	0.1	0.4	0.7	0.1	0.4	0.7
**Gas flow volume: 150 m^3^/h**	**Average value (nm)**	65.99	93.04	146.29	87.65	188.13	192.11	267.38	313.57	278.00
	**Standard deviation (nm)**	24.10	38.46	74.03	43.85	83.61	88.24	70.52	107.22	219.76
	**Confidence interval (nm)**	4.72	7.54	14.51	8.59	16.39	17.30	13.82	21.02	43.04

**Table 3:** Effects of SF concentration and SDBS content on the average diameter of SF nanofibers when the gas flow volume was 50 m^3^/h.

SF (wt %)		6			8			10		
SDBS (wt %)		0.1	0.4	0.7	0.1	0.4	0.7	0.1	0.4	0.7
**Gas flow volume: 50 m^3^/h**	**Average value (nm)**	50.50	78.62	117.07	68.57	103.41	177.78	165.50	295.22	193.55
	**Standard deviation (nm)**	16.51	20.63	50.20	20.63	24.80	56.21	34.01	105.51	47.42
	**Confidence interval (nm)**	3.24	4.04	9.84	4.04	4.86	11.02	6.67	20.68	9.30

**Table 4:** Mechanical properties of SF nanofiber membranes when the gas flow volume was 50 m^3^/h.

SF (wt %)		6			8			10		
SDBS (wt %)		0.1	0.4	0.7	0.1	0.4	0.7	0.1	0.4	0.7
**Average Thickness**	**Average value (μm)**	69.71	61.75	50.67	70.21	69.25	40.00	61.84	65.8	67.30
	**Standard deviation (μm)**	3.31	3.43	4.61	3.33	12.4	9.73	6.21	5.96	2.43
**Breaking Strength**	**Average value (MPa)**	20.73	19.32	18.22	26.15	24.33	23.96	16.97	17.75	20.12
	**Standard deviation (MPa)**	0.64	0.65	0.21	3.75	4.33	11.13	4.66	1.06	0.32
**Elongation at Break**	**Average value (%)**	11.71	13.03	12.48	15.02	16.72	16.93	17.26	18.12	14.01
	**Standard deviation (%)**	1.79	3.23	5.77	5.81	6.17	5.90	1.27	3.70	0.98
