# Correction: Fang et al. High-Throughput Preparation of Silk Fibroin Nanofibers by Modified Bubble-Electrospinning. *Nanomaterials* 2018, *8*, 471

**DOI:** 10.3390/nano12111880

**Published:** 2022-05-31

**Authors:** Yue Fang, Lan Xu, Mingdi Wang

**Affiliations:** 1 National Engineering Laboratory for Modern Silk, College of Textile and Engineering, Soochow University, 199 Ren-ai Road, Suzhou 215123, China; yfang5279@stu.suda.edu.cn; 2 School of Mechanical and Electric Engineering, Soochow University, 178 Ganjiang Road, Suzhou 215021, China

## Error in Figure

In the original publication [1], errors were introduced in two pictures (Figure 5(A-2),(A-3)) in Figure 5 during the copyediting stage. Thus, Figure 5 is incorrect. The authors apologize for any inconvenience caused and state that the scientific conclusions are unaffected. This correction was approved by the Academic Editor. The original publication has also been updated with the correct version of Figure 5, which is also included here.

## Figures and Tables

**Figure 5:**
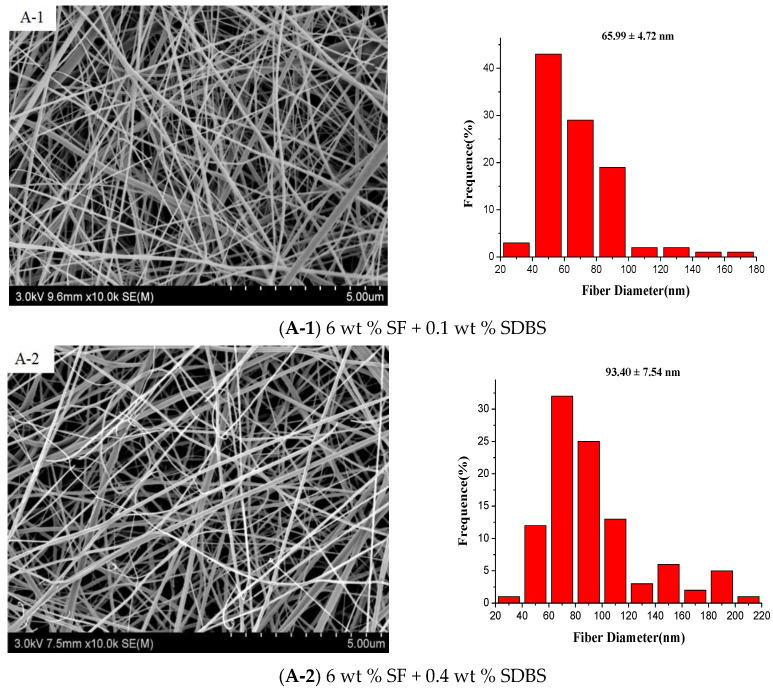

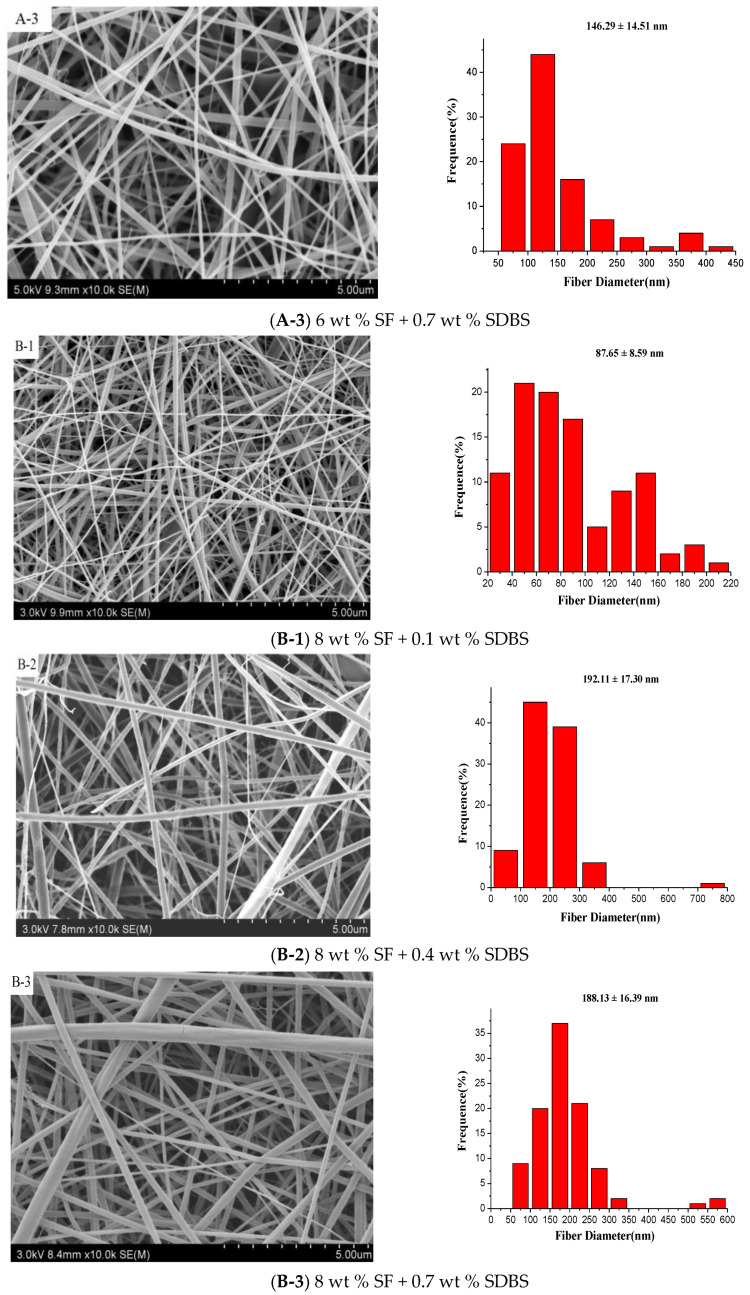

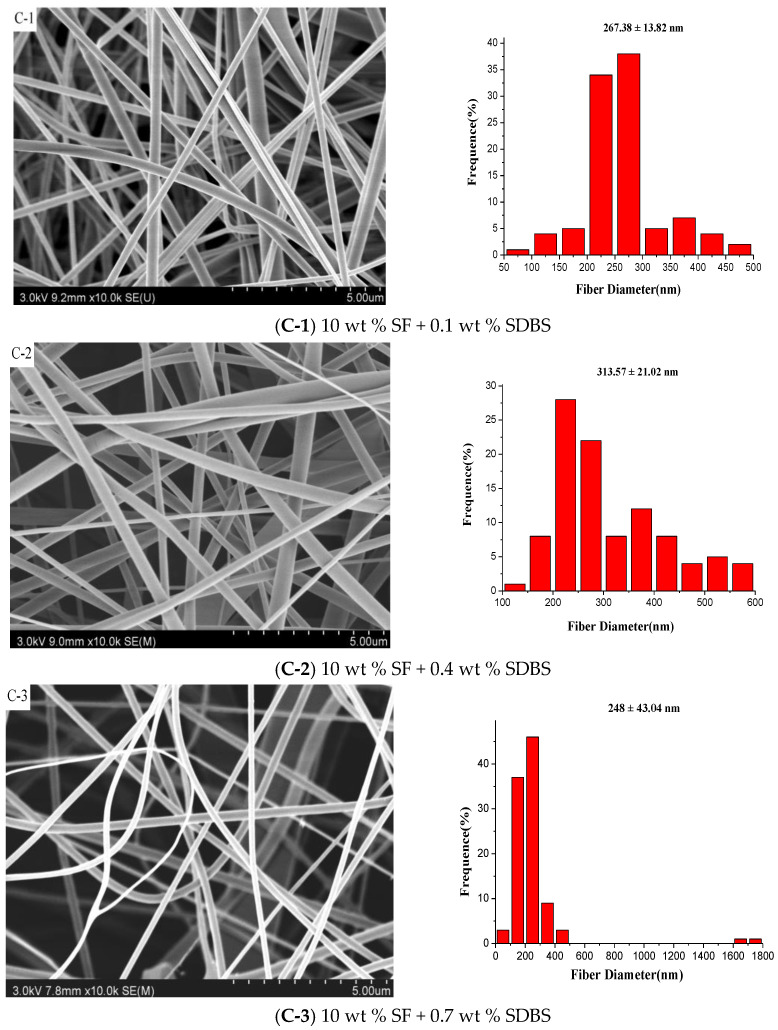
SEM pictures and the according diameter distribution of the SF nanofibers with SF concentrations of (**A**) 6 wt %; (**B**) 8 wt % and (**C**) 10 wt %, respectively. (Gas flow volume: 150 m^3^/h).
